# Copy‐Number Alterations in Central Nervous System Tumours: Diagnostic and Prognostic Implications

**DOI:** 10.1111/nan.70106

**Published:** 2026-11-09

**Authors:** Christian Thomas

**Affiliations:** ^1^ Institute of Neuropathology University Hospital Münster Münster Germany

**Keywords:** copy‐number alterations, molecular classification, neuropathology diagnostics

## Abstract

Copy‐number alterations (CNAs) are frequent molecular events in central nervous system (CNS) tumours ranging from whole‐chromosome gains or losses to focal alterations involving individual genes or regulatory elements. Beyond their biological roles in tumour initiation and progression, distinct CNA patterns have emerged as highly characteristic features of specific CNS tumour types and have been integrated into the WHO classification of CNS tumours as essential diagnostic or grading criteria. Other CNAs provide independent prognostic or predictive information that may inform clinical management. This review provides a comprehensive, practice‐oriented overview of recurrent CNAs across the spectrum of CNS tumours, with emphasis on their diagnostic interpretation in neuropathology. CNAs are classified into a three‐tiered framework: (1) diagnostic CNAs, which define tumour type, molecular subgroup, or CNS WHO grade; (2) prognostic CNAs, which provide independent risk stratification or treatment‐predictive value without directly determining classification or grade; and (3) supportive CNAs, which represent recurrent alterations that may provide important diagnostic clues and help contextualise CNA profiles, but are not yet sufficiently specific or clinically validated for formal diagnostic, grading, or prognostic application. By integrating this concept from molecular neuropathology, this review aims to provide a clinically relevant reference for the interpretation of CNA profiles in neuropathological diagnostics.

## Key Points

- This review classifies the CNAs that frequently occur in CNS tumours, ranging from whole‐chromosome to focal gene‐level events, into a three‐tiered framework: diagnostic, prognostic and supportive.
- Diagnostic CNAs define tumour type, molecular subgroup, or CNS WHO grade, exemplified by combined chr7 gain/chr10 loss in glioblastoma and whole‐arm 1p/19q co‐deletion in oligodendroglioma.
- Prognostic CNAs provide independent risk stratification or treatment‐predictive information without altering tumour classification or grade.
- Most recurrent CNAs across CNS tumour types are supportive rather than formally diagnostic, indicating that their interpretive value often lies in characteristic combinations rather than isolated events.

## Graphical abstract

This review summarises recurrent copy‐number alterations across the spectrum of CNS tumours, classifies them into diagnostic, prognostic and supportive categories, and highlights how characteristic CNA combinations support tumour classification, grading and risk stratification in neuropathological diagnostics.

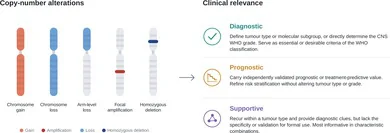

## 1 Introduction

Copy‐number alterations (CNAs) are gains or losses of chromosomal material and are frequently observed in central nervous system (CNS) tumours [1]. Specific CNA patterns are highly characteristic of certain tumour types and have been incorporated in the current WHO classification as diagnostic criteria. Examples include the combined gain of chr7 and monosomy of chr10 in glioblastoma and the whole‐arm co‐deletion of chr1p/19q which is a defining feature of oligodendroglioma [1]. Beyond their specificity for individual tumour types, CNAs also impact grading within a tumour type. For example, a homozygous *CDKN2A/B* deletion warrants designation as CNS WHO grade 4 in IDH‐mutant astrocytoma [2] or CNS WHO grade 3 in meningioma [3], irrespective of histological grading criteria.

## 2 Definition and Mechanisms

CNAs can be classified on a spectrum ranging from entire chromosomes or chromosomal arms to focal changes confined to individual genes or regulatory elements. These categories differ not only in scale but in the biological mechanisms that generate them. At the largest scale, numeric aneuploidy refers to the gain or loss of entire chromosomes and results from errors during cell division, most commonly failure of spindle assembly checkpoint or sister chromatid separation or incorrect attachment of chromosomes to the mitotic spindle [4]. Arm‐level CNAs typically arise from incorrect repair of double‐strand breaks caused by replication stress at structurally vulnerable regions like pericentromeric heterochromatin, telomeres and common fragile sites or mechanical shearing and incorrect sister chromatid splitting [5, 6]. Focal amplifications and deletions emerge from incorrect DNA repair, including error‐prone non‐homologous end joining and replication fork stalling or through the formation of extrachromosomal DNA elements that replicate independently of chromosomes [7, 8]. Unlike the mechanisms described above, chromothripsis involves the catastrophic shattering and error‐prone reassembly of one or a few chromosomes in a single event resulting in multiple adjacent gains and losses [9].

## 3 Detection Methods and Limitations

In neuropathology practice, copy‐number profiling is commonly performed with DNA methylation arrays [10], SNP‐array‐based platforms such as OncoScan or next‐generation sequencing platforms [11, 12]. Although the detection of chromosome‐wide alterations is often straightforward and focal amplifications can even be detected in samples with low tumour content such as infiltration zones of diffuse gliomas [10], focal deletions such as *CDKN2A/B* are more challenging to detect [12, 13]. In a TCGA lower‐grade glioma cohort, for example, conumee 2.0 applied to Illumina 450 k data detected 61% (14/23) of homozygous CDKN2A/B deletions called from matched SNP 6.0 arrays, at a specificity of 99% [13]. Zygosity status is usually defined using arbitrary log2‐ratio cutoff values [14]. However, copy‐number profiles alone cannot reliably distinguish a homozygous deletion in a sample with low tumour content from a heterozygous deletion in a sample with high tumour content, because both may produce similar log2 ratios. Unlike SNP‐based platforms, methylation arrays provide no allele‐specific data to help estimate purity and ploidy. In ambiguous cases, fluorescence in situ hybridisation (FISH) can corroborate locus loss and distinguish single‐copy from homozygous deletion by enumerating locus‐specific signals relative to a reference probe. This distinction requires validated, assay‐specific cut‐off values that account for nuclear truncation in tissue sections and FISH reports copy number per nucleus rather than allelic state [15]. Importantly, CNA calls are not equivalent across platforms. In targeted gene panels in particular, resolution depends on probe density and the distribution of off‐target reads, the copy‐number calling algorithm and its thresholds and tumour cell content [12]. It should therefore be ensured that the technology used is appropriate for the alteration in question. Even then, interpretation remains challenging: a recent international proficiency test of EPIC methylation profiling showed highly reproducible tumour classification but substantial interlaboratory discrepancies in the interpretation of diagnostically relevant CNAs, highlighting the need for harmonised CNA reporting [16]. For gene amplifications, additional pre‐analytical and technical pitfalls include sample swaps in batch‐processed assays, DNA carryover in methylation array workflows [10] and index bleeding in next‐generation sequencing experiments [17, 18]. Many CNAs arise from structural rearrangements and can therefore serve as surrogate markers of underlying gene fusions. RNA or whole‐genome sequencing remains the methods of choice to identify such events. However, rearrangements that activate oncogenes through enhancer hijacking without generating a fusion transcript [19] may escape RNA‐based detection; in such cases, characteristic surrogate CNAs provide a valuable safety net.

## 4 Classification of CNAs According to Diagnostic and Clinical Relevance

Given the broad range of CNA types, their differing biological origins and the variable sensitivity with which they can be detected across diagnostic platforms, interpretation of copy‐number profiles requires more than recognition of recurrent gains and losses. In routine neuropathological diagnostics, the key question is whether a given CNA has direct diagnostic, grading, prognostic, or merely supportive relevance. A previous review by the Cancer Genomics Consortium annotated recurrent CNAs according to their role in the 2016 WHO classification, but without consistently separating them into clinically orientated categories [20]. To provide a practical framework for clinical interpretation adapted to the 2021 WHO classification, this review organises CNAs into three functional categories:

1. **Diagnostic CNAs:** Copy‐number changes that define tumour type or molecular subgroup, serving as diagnostic markers or directly affecting the CNS WHO tumour grade.
2. **Prognostic CNAs:** Alterations with independently validated prognostic or treatment‐predictive value (grouped here for simplicity), informing patient risk stratification and treatment selection, but without direct impact on tumour type or grading.
3. **Supportive CNAs:** Recurrent copy‐number changes that are frequently observed within a given tumour type and may provide useful diagnostic clues or help contextualise CNA profiles, but lack the specificity or clinical validation currently required for formal diagnostic, grading, or prognostic interpretation (e.g., chr10 loss in papillary tumour of the pineal region). This category also includes CNAs with preliminary outcome associations that have not yet been independently validated.

Using this classification scheme, the following sections systematically review recurrent and clinically relevant CNAs across major CNS tumour categories, including adult‐type diffuse gliomas, paediatric‐type diffuse gliomas, circumscribed astrocytic tumours, ependymal tumours, glioneuronal and neuronal tumours, embryonal tumours, meningiomas, pineal tumours and other relevant CNS neoplasms. The aim is to provide a structured reference for the interpretation of copy‐number profiles in routine neuropathological diagnostics and neuro‐oncological decision‐making.

## 5 Adult‐Type Diffuse Gliomas

In astrocytoma, IDH‐mutant, the most important diagnostic CNA is homozygous deletion of *CDKN2A/B*, which warrants designation as CNS WHO grade 4 irrespective of histological features [21, 22, 23] (Figure 1). Importantly, heterozygous *CDKN2A/B* deletion does not appear to confer equivalent prognostic significance, especially after adjusting for clinical parameters [14, 24]. Several prognostic CNAs have been associated with adverse outcome in IDH‐mutant astrocytoma, including homozygous *RB1* deletion, *CDK4* amplification, *PDGFRA* amplification, *MET* amplification, *MYCN* amplification and chr14q loss [22, 23, 25, 26, 27]. Additionally, the total CNV load is correlated with tumour grade and adverse prognosis in IDH‐mutant astrocytoma [23, 28]. Other recurrent CNAs include amplifications of *MYC* and *CCND2*, which have been reported in comprehensive genomic profiling studies, but their independent diagnostic or prognostic relevance remains uncertain [23, 29].

**FIGURE 1:**
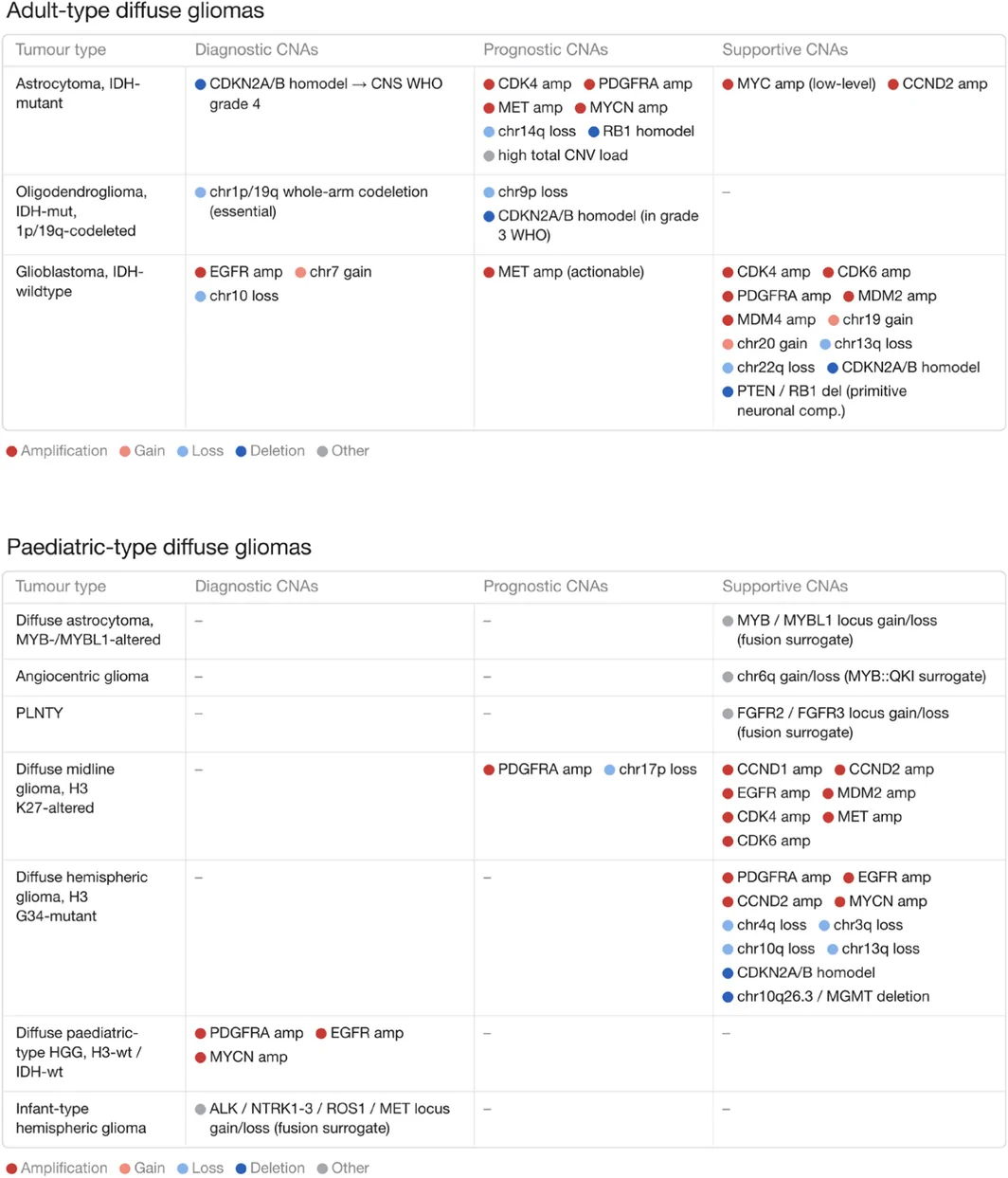
Recurrent copy‐number alterations in adult‐type diffuse gliomas and paediatric‐type diffuse gliomas.

In oligodendroglioma, IDH‐mutant and 1p/19q‐codeleted, whole‐arm loss of chr1p and 19q is an essential diagnostic criterion and its absence precludes the diagnosis [1, 30]. Additional CNAs may further refine prognostic assessment within this entity. In particular, chr9p loss has been associated with shortened survival [31] and superimposed homozygous *CDKN2A/B* deletion in grade 3 oligodendrogliomas is associated with particularly poor outcome [32].

In glioblastoma, IDH‐wildtype, the most characteristic diagnostic CNA pattern consists of combined chr7 gain and chr10 loss, frequently accompanied by *EGFR* amplification [1]. Although several partial chr7/10 configurations have been described, only combined whole chr7 gain and whole chr10 loss is a formal diagnostic criterion. In a cohort of 939 IDH‐wildtype gliomas, Stichel et al. found that (i) whole chr7 gain with whole chr10 loss, (ii) whole chr7 gain with isolated chr10q loss and (iii) chr7q gain with whole chr10 loss were associated with a survival similar to that of IDH‐wildtype glioblastoma and proposed these as a ‘7/10 signature’ [33]. However, the partial configurations were rare (*n* = 9 and 12), the analysis was univariable and rarer combinations could not be evaluated. Several additional CNAs reflect the core oncogenic pathways disrupted in glioblastoma, including cell‐cycle deregulation through *CDK4* or *CDK6* amplification and homozygous *CDKN2A/B* deletion, RTK/PI3K pathway activation through *PDGFRA* amplification and p53 pathway disruption through *MDM2* or *MDM4* amplification [34]. Recurrent chromosome 13q and 22q losses, as well as chromosome 19 and 20 gains, further contribute to the broader copy‐number landscape of glioblastoma [35]. Among prognostically and therapeutically relevant alterations, *MET* amplification has been associated with poor outcome and may represent an actionable target in selected cases [36]. Finally, homozygous *PTEN* and *RB1* deletions are enriched in glioblastomas with a primitive neuronal component, a histological pattern associated with a distinct DNA methylation and copy‐number profile [37].

## 6 Paediatric‐Type Diffuse Gliomas

Among paediatric‐type diffuse gliomas, copy‐number changes often reflect the underlying oncogenic driver rather than serving as independent diagnostic or prognostic markers (Figure 1).

In diffuse astrocytoma, *MYB*‐ or *MYBL1*‐altered, focal copy‐number gains or losses at the *MYB* and *MYBL1* loci are linked to the gene fusions that define these indolent tumours [38]. Similarly, in angiocentric glioma, a gain or loss at chr6q is a surrogate marker for the *MYB::QKI* fusion that underlies this entity [39, 40].

In polymorphous low‐grade neuroepithelial tumour of the young (PLNTY), gains or losses involving *FGFR2* and *FGFR3* loci are alterations reflecting underlying kinase pathway fusions, though they lack established diagnostic or prognostic value [41].

In diffuse midline glioma, H3 K27‐altered, *PDGFRA* amplification and loss of chr17p are the most clinically relevant CNA and predict poor prognosis [42, 43]. Other high‐level amplifications (*CCND1*, *CCND2*, *EGFR*, *MDM2*, *CDK4*, *CDK6*, *MET*) were observed in single cases only [42, 44].

In diffuse hemispheric glioma, H3 G34‐mutant, the copy‐number landscape is characterised predominantly by broad chromosomal imbalances, most commonly involving loss of chr4q (70%), chr3q (67%), chr10q (60%) and chr13q (57%) as well as *PDGFRA* amplification (27%) [45]. Other focal events are homozygous deletions of *CDKN2A/B* and deletions of chr10q26.3 involving the *MGMT* locus as well as amplifications of *EGFR*, *CCND2*, *MYCN* [46]. None of these alterations currently carry established prognostic significance independent of the defining H3 G34 mutation.

In diffuse paediatric‐type high‐grade glioma, H3‐wildtype and IDH‐wildtype, *PDGFRA*, *EGFR*, or *MYCN* alterations (including amplifications) characterise the pHGG RTK1, RTK2 and MYCN subtypes [47]. According to the current WHO classification, in a diffuse glioma with mitotic activity in a child or young adult lacking IDH1/2 and H3 mutations, these alterations can establish the diagnosis as an alternative to a matching methylation profile.

In infant‐type hemispheric glioma, focal gains or losses at *ALK*, *NTRK1*‐*3*, *ROS1* and *MET* loci are surrogate markers for the oncogenic kinase fusions that define this entity and represent potential therapeutic targets [48].

## 7 Circumscribed Astrocytic Gliomas

Focal structural variants with low‐level gains and losses of the *BRAF* locus on chr7q34 are indicative of *BRAF* fusions in pilocytic astrocytoma [10] (Figure 2). Whereas focal gains typically reflect the tandem duplication generating the canonical *KIAA1549::BRAF* fusion, partial deletions may indicate alternative rearrangements such as *FAM131B::BRAF*. In addition, whole‐chromosome gains, most frequently of chr5 and chr7, are observed in a subset of cases, particularly in adults and represent supportive CNAs [49, 50]. In contrast, segmental or complex copy‐number alterations, in particular homozygous *CDKN2A/B* deletion, should prompt consideration of alternative diagnoses such as high‐grade astrocytoma with piloid features.

**FIGURE 2:**
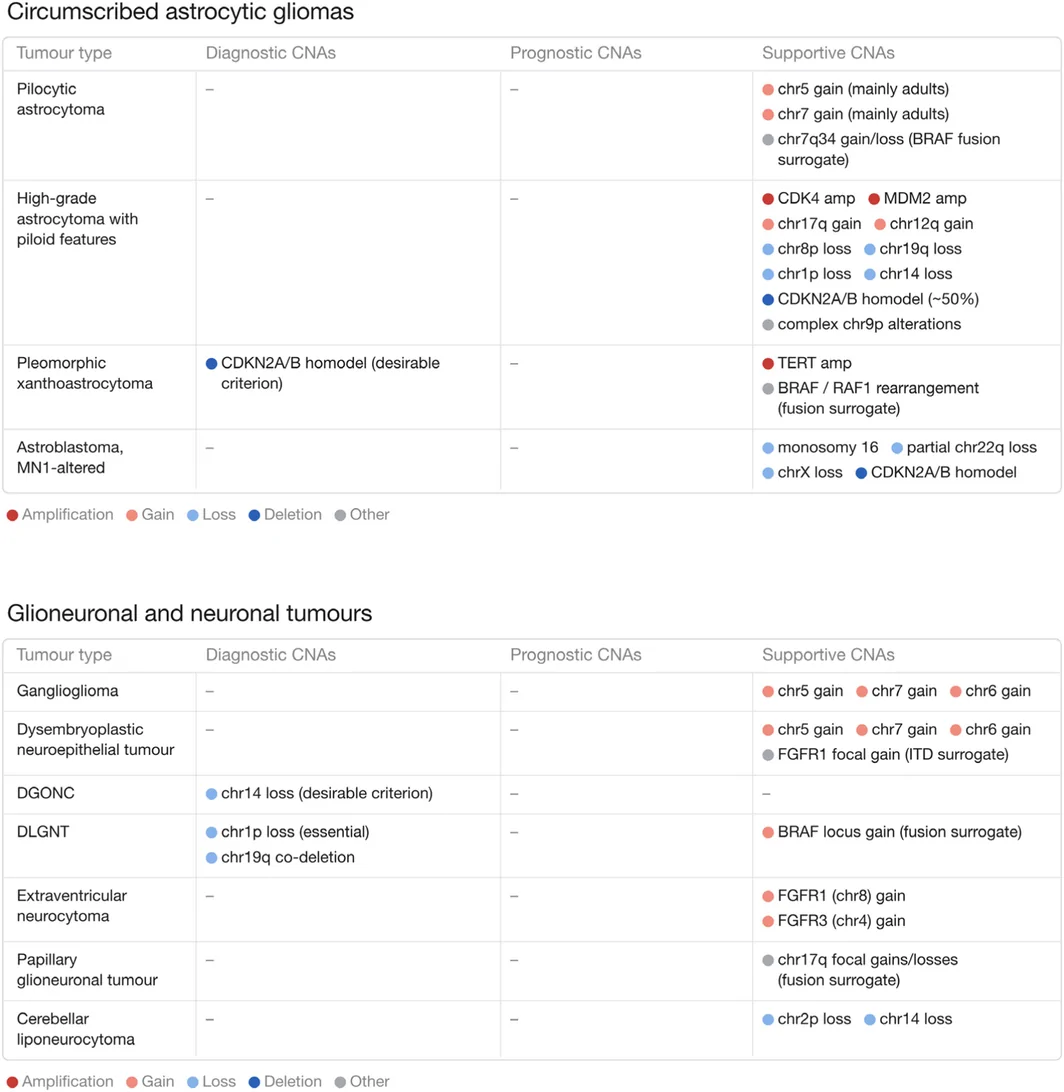
Recurrent copy‐number alterations in circumscribed astrocytic gliomas and glioneuronal and neuronal tumours.

High‐grade astrocytoma with piloid features (HGAP) is most commonly characterised by homozygous *CDKN2A/B* deletion, often occurring in the context of broader or complex chr9p alterations and present in nearly half of cases [51]. Additional recurrent copy‐number changes include gains or complex alterations of chr17q and chr12q, as well as losses of chr8p, chr14, chr19q and chr1p, whereas *CDK4* and *MDM2* amplifications are less frequent [51].

In pleomorphic xanthoastrocytoma (PXA), homozygous deletion of *CDKN2A/B* is observed in most cases and thus represents a desirable diagnostic CNA criterion. Less frequent structural alterations include rearrangements involving *BRAF* and *RAF1* (indicative of kinase fusions) as well as *TERT* amplification [52].

In astroblastoma, MN1‐altered, recurrent CNAs include homozygous deletions of *CDKN2A/B*, monosomy of chr16 and partial losses of chr22q and chrX, probably reflecting the chromosomal rearrangement process leading to *MN1::BEND2* fusion [53, 54, 55, 56]. However, none of these changes currently carry established diagnostic or prognostic relevance.

Subependymal giant cell astrocytoma and chordoid glioma of the third ventricle do not exhibit characteristic copy‐number alterations, consistent with their predominantly mutation‐driven pathogenesis (*TSC1/TSC2* and *PRKCA* mutations).

## 8 Glioneuronal and Neuronal Tumours

Most glioneuronal and neuronal tumours are defined primarily by molecular alterations other than copy‐number changes and several entities, including gangliocytoma, desmoplastic infantile ganglioglioma/desmoplastic infantile astrocytoma, rosette‐forming glioneuronal tumour, myxoid glioneuronal tumour, multinodular and vacuolating neuronal tumour, dysplastic cerebellar gangliocytoma and central neurocytoma do not exhibit recurrent diagnostic or prognostically relevant copy‐number alterations.

Among entities with characteristic CNA findings, diffuse glioneuronal tumour with oligodendroglioma‐like features and nuclear clusters (DGONC) is notable for chr14 loss, which is observed in the majority of cases and represents a desirable diagnostic criterion [57] (Figure 2).

In ganglioglioma and dysembryoplastic neuroepithelial tumour, whole‐chromosome gains, most frequently of chr5, chr7 and chr6, represent recurrent supportive CNAs; of note, chr5 gain was not observed in diffuse low‐grade astrocytomas and may thus aid the differential diagnosis [58]. In dysembryoplastic neuroepithelial tumour, intragenic duplication of the *FGFR1* tyrosine kinase domain (*FGFR1* ITD) is a frequent driver alteration [59] that may appear as a small focal gain at the *FGFR1* locus (chr8p11.23). Its detection depends on platform resolution and tumour cell content and targeted methods such as NGS, MLPA, or digital PCR are more reliable [60, 61].

In diffuse leptomeningeal glioneuronal tumour (DLGNT), chr1p loss is a highly recurrent finding and thus an essential diagnostic criterion in the WHO classification, sometimes by a co‐deletion with chr19q [62]. In addition, focal *BRAF* gains reflecting frequent *BRAF* rearrangements may also aid the diagnosis [62].

In extraventricular neurocytoma, focal gains at *FGFR1* (chr8) and *FGFR3* (chr4) loci are supportive for the diagnosis as surrogate markers of the recurrent *FGFR1::TACC1* and related fusions that characterise this entity [63].

Papillary glioneuronal tumour may show flat genomes or focal chr17q gains/losses at the *PRKCA* locus associated with the canonical *SLC44A1::PRKCA* fusion [64].

Cerebellar liponeurocytoma shows recurrent focal losses at chr2p and chr14 without established diagnostic or prognostic significance [10].

## 9 Ependymal Tumours

In ependymal tumours, copy‐number profiles provide both diagnostic and prognostic information that varies substantially across the anatomically and molecularly distinct subtypes (Figure 3).

**FIGURE 3:**
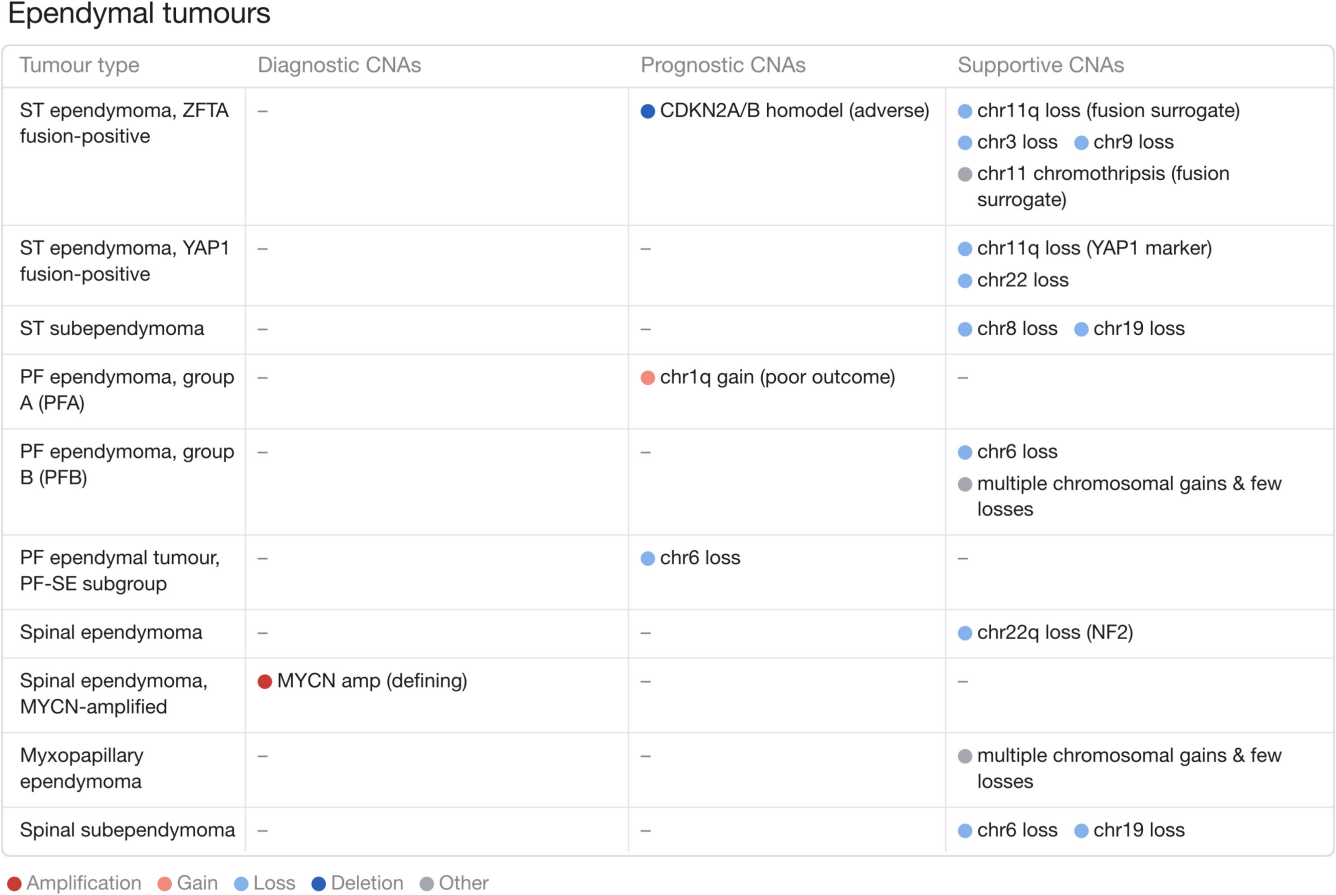
Recurrent copy‐number alterations in ependymal tumours.

In supratentorial ependymoma, *ZFTA* fusion‐positive, chromothripsis of chr11 or focal losses on chr11q are associated with the prototypical *ZFTA::RELA* or related fusions [65]. Other characteristic genomic losses in this entity involve chr3 and chr9 [10, 66]. Homozygous *CDKN2A/B* deletion is a prognostically adverse event associated with shortened survival in this entity [67]. In supratentorial ependymoma, *YAP1* fusion‐positive, a focal chr11q loss is a relevant marker of the underlying *YAP1* fusion, whereas recurrent chr22 loss can be observed in an otherwise flat CNA profile [10]. In line with their benign nature, supratentorial subependymomas show a relatively quiet genome with losses of chr8 and chr19 being the only recurrent chromosomal changes [66].

In posterior fossa group A (PFA) ependymoma, gain of chr1q is the most established prognostic CNA, consistently associated with poor outcome across multiple independent cohorts [68, 69]. In contrast, posterior fossa group B (PFB) ependymomas show a complex genomic background with multiple recurrent whole‐chromosomal gains and few losses, most frequently affecting chr6 [10, 70]. In posterior fossa ependymal tumours of the PF‐SE molecular subgroup (which mainly comprise subependymomas, but also mixed subependymoma‐ependymoma or pure ependymoma phenotypes on histology), chr6 loss (together with *TERT* promoter mutations) is associated with a higher tendency of recurrences [71].

For spinal ependymoma, chr22q loss is the most prevalent finding reflecting the underlying pathobiology with co‐occurring *NF2* mutations [72]. Spinal ependymoma, *MYCN*‐amplified, is a distinct aggressive variant in which *MYCN* amplification is the defining diagnostic criterion [73]. Myxopapillary ependymomas show a complex karyotype with multiple recurrent gains and (fewer) losses [66]. In spinal subependymomas, losses of chr6 and chr19 are the only relevant CNAs [66, 72].

## 10 Embryonal Tumours

Copy‐number alterations carry major diagnostic and prognostic significance in embryonal tumours, particularly in medulloblastoma (Figure 4). Stereotypical CNAs are strongly associated with medulloblastoma molecular subgroups.

**FIGURE 4:**
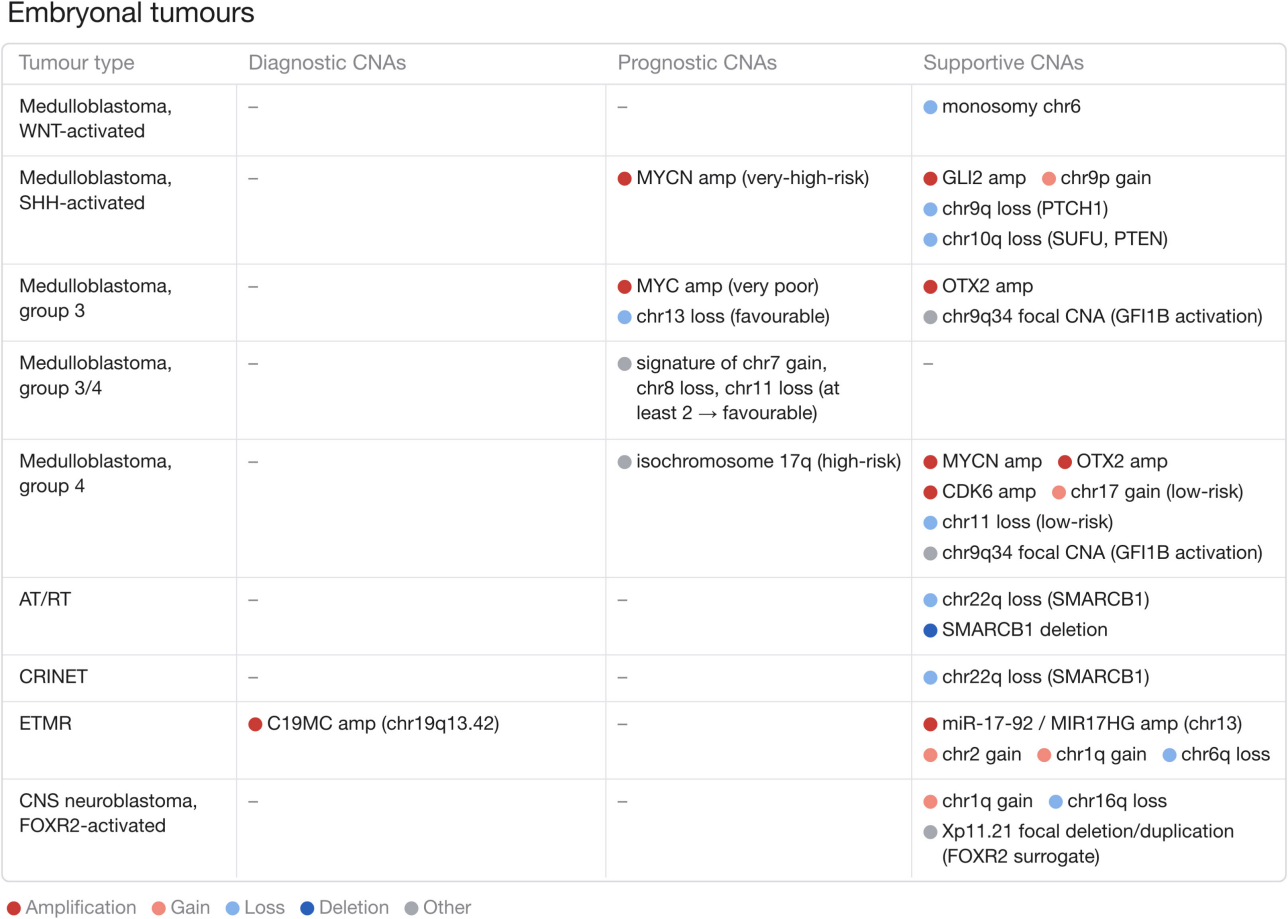
Recurrent copy‐number alterations in embryonal tumours.

Monosomy of chr6 is characteristic of the prognostically favourable WNT subgroup [74, 75], although it is not entirely specific, as it can also occur in a proportion of SHH and group 4 tumours, particularly in adults [76].

In SHH medulloblastomas, infants show few recurrent chromosomal CNAs and the defining molecular associations are mutational (*SUFU*, *PTCH1*) rather than copy‐number [75]; however, loss of chr10q, encompassing *SUFU* and *PTEN*, frequently provides the second hit in *SUFU*‐mutant infant tumours [77]. In older children with SHH medulloblastoma, loss of chr9q (encompassing the *PTCH1* locus), frequently together with gain of chr9p, as well as *GLI2* and *MYCN* amplifications, are observed [75]. Of note, *MYCN* amplification, LCA pathology and *TP53* mutation frequently co‐occur and define the very‐high‐risk SHH phenotype [75].

In the non‐WNT/non‐SHH groups, *MYC* amplification is the characteristic group 3 amplicon and confers a very poor prognosis, whereas chr13 loss predicts improved outcome [75]. *OTX2* amplification is observed in a subset of group 3 and group 4 tumours [74]. In addition, focal copy‐number changes at chr9q34 may indicate structural variants activating *GFI1B* through enhancer hijacking in group 3 and, less frequently, group 4 tumours, whereas *GFI1* activation is mostly caused by translocations without detectable copy‐number change [19]. A combined CNA signature, defined as at least two of chr7 gain, chr8 loss and chr11 loss, identifies a favourable‐risk non‐WNT/non‐SHH group in trial‐treated patients [78]; of note, isolated chr7q gain has conversely been reported as an adverse factor within group 4 [75], consistent with this high‐ploidy balanced signature behaving differently from individual gains. Isochromosome 17q (manifesting as loss of chr17p and gain of chr17q) is a hallmark event of group 4, enriched specifically in the high‐risk subgroup, although it was not independently prognostic in multivariate analysis; chr11 loss and chr17 gain are associated with low‐risk group 4 tumours [75]. *MYCN* amplification, by contrast, is the typical amplicon of group 4 (and SHH), is detectable only rarely in group 3 and (unlike in SHH) does not confer an adverse prognosis in group 4 [75]. *CDK6* amplification is likewise enriched in group 4 [74].

In atypical teratoid/rhabdoid tumour (AT/RT), biallelic inactivation of *SMARCB1* on chr22q is the molecular hallmark of this entity, though the underlying mechanism is subgroup‐dependent [79]: ATRT‐TYR most often shows monosomy 22 (77%), ATRT‐MYC focal *SMARCB1* deletions (79%) and ATRT‐SHH frequently no detectable chr22 copy‐number change (48%), implicating point mutations or small indels [79]. As in ATRT‐TYR, monosomy 22 is also the typical alteration in cribriform neuroepithelial tumour (CRINET), reflecting *SMARCB1* inactivation shared with AT/RT but occurring in a distinct epigenetic context [80].

In embryonal tumour with multilayered rosettes (ETMR), amplification of a microRNA cluster, most commonly C19MC on chromosome 19q13.42, is present in approximately 90% of cases and represents both the genetic hallmark and an essential diagnostic criterion. Rarely, the miR‐17‐92 cluster, encoded by MIR17HG on chromosome 13, is amplified instead. Cases lacking either amplification are enriched for biallelic *DICER1* mutations [81]. Gain of chr2 is the most frequent accompanying copy‐number aberration (~70%) followed by chr1q gain (~25%) and chr6q loss (~20%) [81].

Frequent copy‐number changes in CNS neuroblastoma, *FOXR2*‐activated, are gains of chr1q in nearly all cases and chr16q loss (70% of cases) [10, 82]. In a subset of cases, focal deletions or duplications at chrXp11.21 reflect the structural rearrangements that activate *FOXR2*, most frequently a deletion extending approximately 500 kb upstream of the gene [53]. However, activating events such as translocations, inversions, or small insertions may not cause detectable copy‐number changes [53] and chrX is often not displayed in standard methylation array‐based CNV plots.

CNS tumour with BCOR internal tandem duplication is not associated with recurrent copy‐number alterations [10].

## 11 Meningioma

Copy‐number profiling informs both grading and subtyping of meningiomas (Figure 5). According to the current WHO CNS5 classification, the most consequential alteration is homozygous deletion of *CDKN2A/B*, which designates CNS WHO grade 3 irrespective of histology because it independently predicts early recurrence and shortened survival [3, 83]. Building on this principle, cIMPACT‐NOW Update 8 proposed that deletion of chr1p (either complete or segmental, i.e., > 5% of the arm) combined with chr22q deletion and/or an *NF2* pathogenic variant upgrades an otherwise histologically benign meningioma to CNS WHO grade 2, although this is not an official WHO CNS5 grading criterion [3]. Monosomy 22 is the most frequent and earliest event, present in over half of meningiomas and tightly linked to *NF2* inactivation; it constitutes the founding aberration from which further imbalances accumulate, with *NF2*‐mutant tumours showing the greatest genomic instability [3, 84]. Loss of chr1p typically follows and is the single most consistent marker of aggressive behaviour, exceeding the frequency of other recurrent losses on chr6p/q, chr10q, chr14q and chr18p/q that become enriched in higher‐grade tumours [3, 83]. Reflecting the prognostic weight of these patterns, genome‐wide copy‐number– and methylation‐based models stratify recurrence risk more accurately than histological grading alone and underpin integrated molecular–morphological grading schemes [85, 86]. Chromosomal gains are less common and prognostically heterogeneous: gain of chr1q occurs in high‐grade tumours, whereas whole‐chromosome gains, most often chr5 and chr7, characterise the indolent angiomatous, metaplastic and microcystic subtypes [83].

**FIGURE 5:**
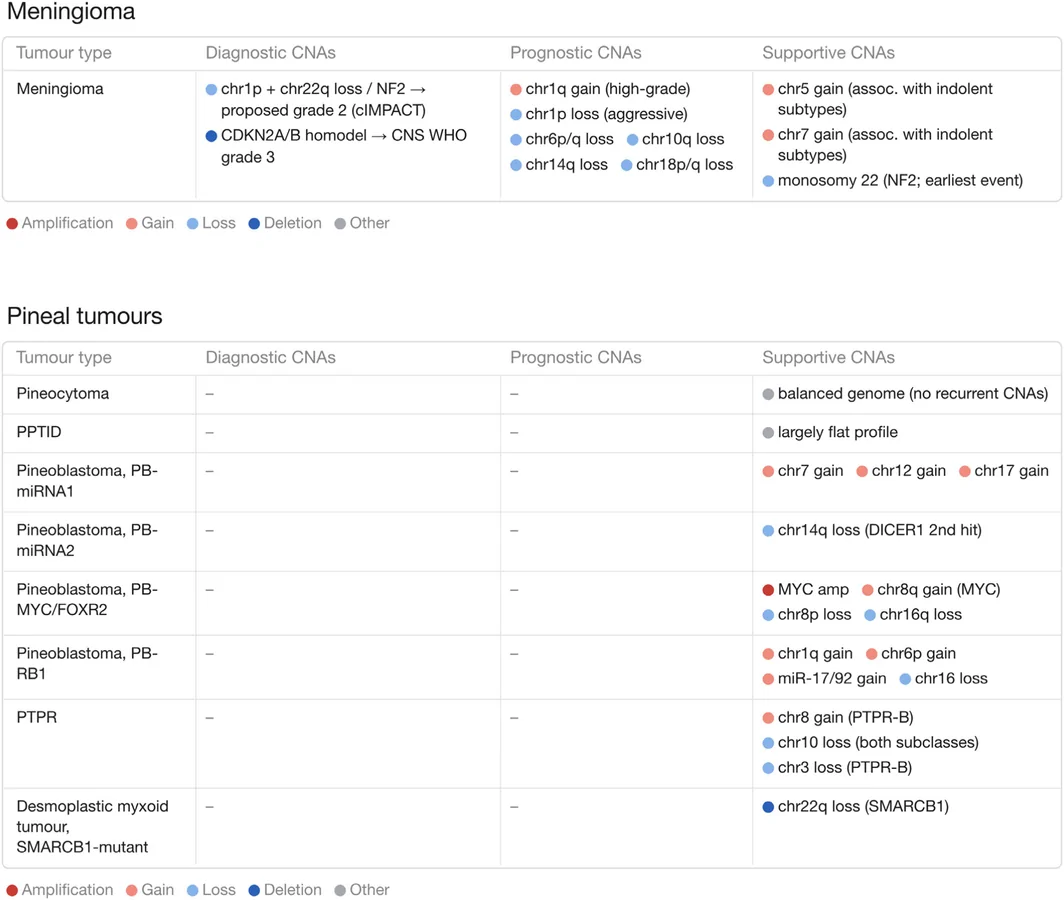
Recurrent copy‐number alterations in meningioma and pineal tumours.

## 12 Pineal Tumours

The copy‐number landscape of pineal parenchymal tumours is strongly entity‐ and grade‐dependent (Figure 5). Pineocytoma characteristically displays a balanced genome devoid of recurrent imbalances [87]. Pineal parenchymal tumour of intermediate differentiation (PPTID) likewise shows largely flat profiles, with only sporadic broad gains or losses of uncertain significance [10, 88].

Pineoblastomas (PB) comprise four molecular subgroups with distinct CNA landscapes [89]. The two miRNA‐processing‐driven subgroups differ cytogenetically: PB‐miRNA1 shows arm‐level gains of chr7, chr12 and chr17, whereas PB‐miRNA2 is characterised by chr14q loss, which constitutes the second‐hit mechanism for complete inactivation of *DICER1*. PB‐MYC/FOXR2 tumours show recurrent broad chr8p and chr16q loss, with chr8q gain encompassing *MYC* in 18% and focal *MYC* amplification in 6% of cases. PB‐RB1 is defined by recurrent chr1q and chr6p gain together with chr16 loss, mirroring the cytogenetic profile of retinoblastoma; focal *miR‐17/92* gain occurs as an additional recurrent event in approximately 25% of cases.

Papillary tumours of the pineal region (PTPR) can be divided into two distinct methylation subclasses (PTPR‐A and PTPR‐B), both of which very frequently show chr10 loss [88]. Of clinical importance, isolated chr10 loss in PTPR‐B has been associated with *PTEN* germline mutations, whereas sporadic PTPR‐B frequently show other CNAs including loss of chr3 and gain of chr8 [90]. Desmoplastic myxoid tumour of the pineal region, *SMARCB1*‐mutant, deletion of the chr22q/*SMARCB1* locus might occur, reflecting the SWI/SNF inactivation shared across *SMARCB1*‐deficient CNS tumours [91].

## 13 Other CNS Tumours

Other CNS tumour categories, including sellar region, choroid plexus, germ cell, melanocytic, lymphoid, vascular and mesenchymal neoplasms, each display characteristic CNA profiles summarised below and in Figure 6.

**FIGURE 6:**
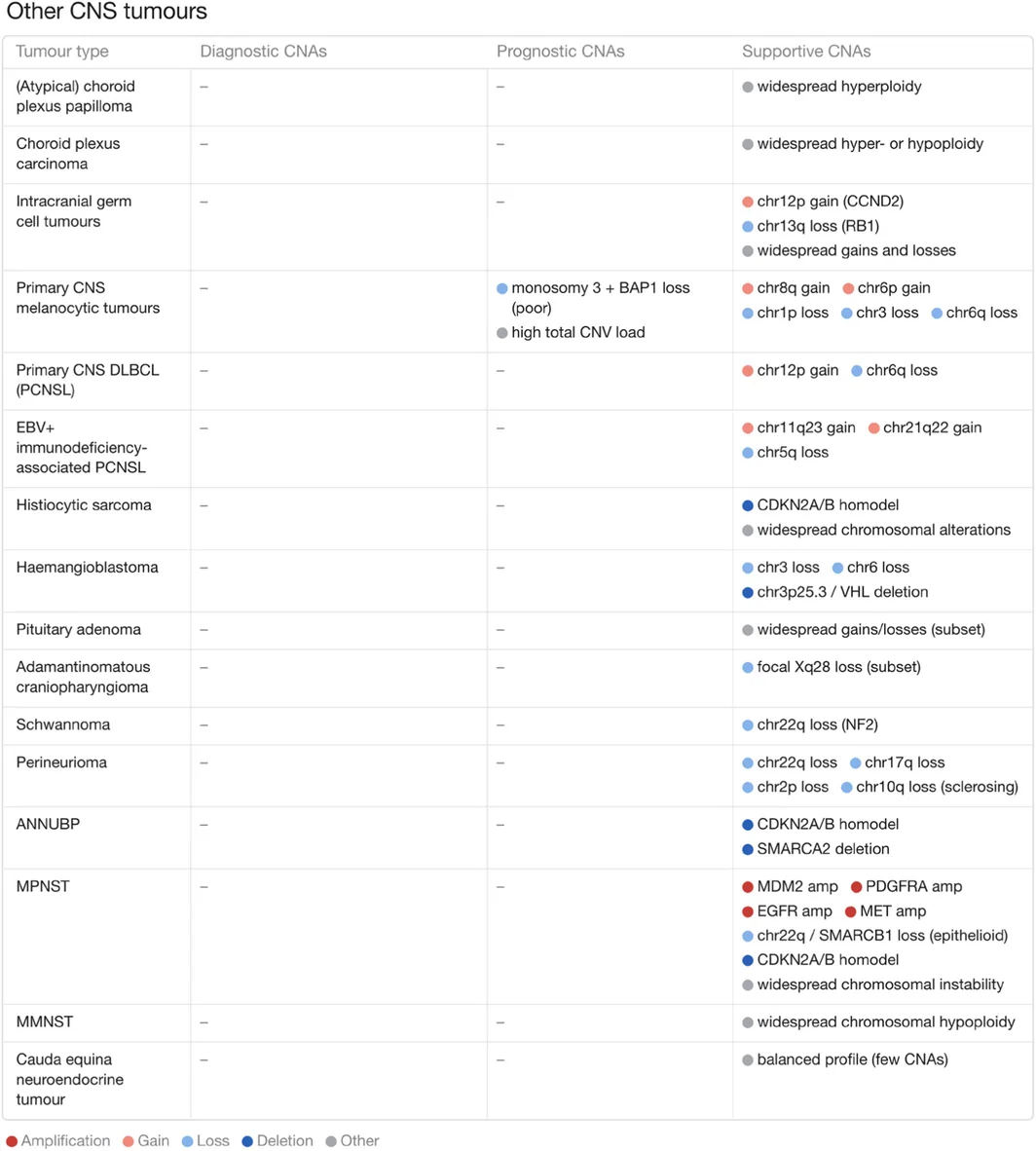
Recurrent copy‐number alterations in other CNS tumours.

### 13.1 Choroid Plexus Tumours (CPTs)

Among CPTs, papillomas and atypical papillomas share a pattern of widespread chromosomal hyperploidy with largely overlapping profiles between the two grades, whereas choroid plexus carcinomas may show either widespread hyper‐ or hypoploidy [92]. Thus, a CPT with a flat CNV profile should raise suspicion and alternative diagnoses, such as normal choroid plexus or choroid plexus hyperplasia should be considered [93].

### 13.2 Intracranial Germ Cell Tumours

Similar to CPTs, intracranial germ cell tumours display widespread gains and losses, with recurrent gain of chr12p (*CCND2*) and loss of chr13q (*RB1*), implicating relevance of the cyclin/CDK–RB–E2F pathway [94].

### 13.3 Melanocytic Tumours

Primary melanocytic neoplasms of the CNS encompass melanocytosis, melanomatosis, melanocytoma and primary leptomeningeal melanoma. Because histology correlates poorly with clinical behaviour, CNAs have gained diagnostic and prognostic relevance. The genome‐wide burden of large CNAs tracks malignancy and disease progression, increasing as disease advances even in histologically bland lesions, whereas indolent, stable lesions may show a near‐normal profile, although these observations rest on small case numbers [95, 96]. Many of these tumours share a copy‐number profile with uveal melanoma and blue nevus‐like melanoma, with gains of chr8q and chr6p and losses of chr1p, chr3 and chr6q, which distinguishes them from cutaneous melanoma metastases and defines a poor‐prognosis subset marked by monosomy chr3 and BAP1 loss [97].

### 13.4 Hematolymphoid Tumours

Primary CNS diffuse large B‐cell lymphoma (PCNSL) harbours multiple CNAs, with chr6q loss and chr12p gains each occurring in more than half of cases [10]. EBV + immunodeficiency‐associated PCNSL shows a distinct profile displaying recurrent gains on chr11q23, a locus directly targeted for genomic aberrations by the EBV protein EBNA1 and on chr21q22, alongside losses on chr5q [98].

Histiocytic sarcoma of the CNS shows widespread chromosomal alterations and frequent *CDKN2A/B* deletion, reflecting its complex genomic landscape [99].

### 13.5 Hemangioblastomas

Hemangioblastomas are characterised by either whole chr3 loss or focal chr3p25.3 deletion encompassing the *VHL* locus, alterations that occur in both sporadic and von Hippel–Lindau syndrome‐associated tumours [100]. Less frequent copy‐number changes include loss of chr6.

### 13.6 Tumours of the Sellar Region

In pituitary adenoma/pituitary neuroendocrine tumour, widespread gains and losses relate to hormone secretion status, with disrupted profiles predominantly observed in functioning tumours [101]. Granular cell tumour, pituicytoma and spindle cell oncocytoma do not show recurrent CNAs [10]. In adamantinomatous craniopharyngioma, a subset of cases shows recurrent focal chromosome Xq28 loss whereas others show focal gains or stable genomes, whereas papillary craniopharyngioma has stable copy‐number profiles, consistent with its predominantly BRAF V600E‐driven biology [102].

### 13.7 Nerve Sheath Tumours

In schwannoma, chr22q loss encompassing the *NF2* locus is the most recurrent event in both sporadic and *NF2*‐associated cases [103], whereas perineurioma shows recurrent (and typically mutually exclusive) losses of chr22q (*NF2*) and chr17q (*NF1*) [104]. Within the neurofibroma spectrum, homozygous *CDKN2A/B* and heterozygous *SMARCA2* deletion mark ANNUBP [105, 106], whereas MPNST is defined by widespread chromosomal instability with amplifications of *MDM2*, *PDGFRA*, *EGFR* and *MET* and homozygous *CDKN2A/B* deletion [106]. Homozygous loss of *SMARCB1* is observed in the epithelioid MPNST variant [107]. MMNST is characterised by widespread chromosomal gains and losses [108, 109].

## 14 Conclusion

Across the broad spectrum of CNS tumours covered in this review, recurrent copy‐number alterations represent a molecular feature with direct relevance for diagnosis, grading and risk stratification. Applying the proposed three‐tiered framework to the 206 recurrent CNAs across the 65 tumour types discussed here, 8% (*n* = 17) carry direct diagnostic or grading relevance and 14% (*n* = 28) provide independent prognostic or predictive information, whereas the remaining 78% (*n* = 161) represent supportive CNAs (Figure 7). Although only a minority of recurrent CNAs currently function as stand‐alone diagnostic or prognostic biomarkers, many supportive alterations contribute substantially to integrated molecular classification when interpreted alongside histological, epigenetic and mutational findings.

**FIGURE 7:**
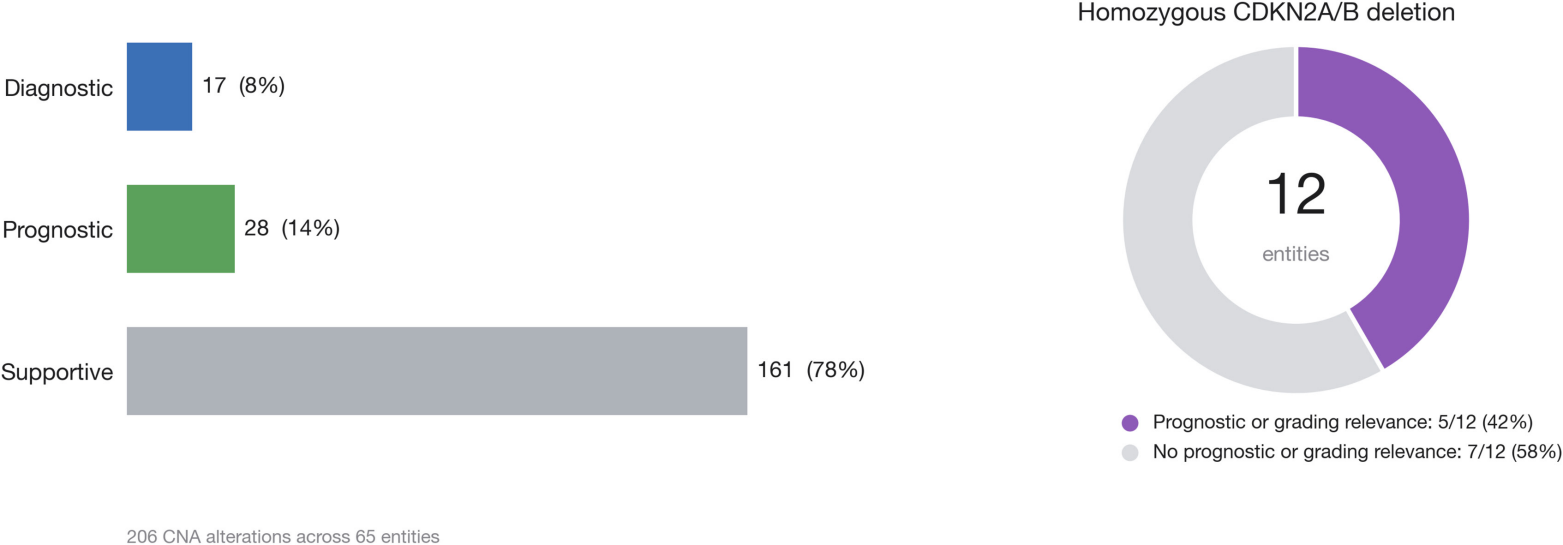
Summary of copy‐number alterations across CNS tumours.

The most widely shared alterations across tumour types are homozygous deletion of *CDKN2A/B* (*n* = 12 tumour types), loss of chr22q encompassing the *NF2* locus (*n* = 7) and amplification of cell‐cycle and receptor tyrosine kinase oncogenes, most notably *MYCN* and *PDGFRA* (*n* = 6 each). Among these, homozygous *CDKN2A/B* deletion represents the single most pervasive recurrent CNA and exemplifies how a focal copy‐number event can carry major diagnostic and prognostic implications. At the same time, proficiency testing demonstrates that accurate determination of focal deletions remains technically challenging [16] and depends on assay characteristics, tumour cell content and interpretation thresholds [14], emphasising the need for quality assurance and cautious interpretation of borderline findings.

Beyond their immediate clinical utility, recurrent CNAs provide important insights into the biological mechanisms driving CNS tumourigenesis, highlighting recurrent themes such as cell‐cycle dysregulation, receptor tyrosine kinase activation, chromatin remodelling and chromosomal instability. Technological advances have further increased their diagnostic relevance. Genome‐wide CNA profiling is now routinely obtained as a by‐product of DNA methylation arrays and increasingly also from targeted NGS panels, without requiring dedicated additional assays. Assessment of allelic imbalance and copy‐neutral loss of heterozygosity, however, depends on platforms with allelic information (e.g., SNP arrays or NGS panels with a SNP backbone) and corresponding analysis pipelines; it is not possible from methylation array intensities alone.

Importantly, the predominance of supportive CNAs identified in this review indicates that the diagnostic value of copy‐number alterations often lies not in isolated events but in characteristic combinations of chromosomal gains and losses interpreted within the broader molecular context. As molecular classification systems continue to evolve, some currently supportive CNAs will likely gain direct diagnostic or prognostic significance, whereas others may improve biological subclassification of established entities. Together, these findings demonstrate that recurrent CNAs are not only secondary genomic alterations but constitute a central layer of information for understanding CNS tumour biology, refining classification and guiding clinical decision‐making in modern neuro‐oncology.

## Author Contributions

Christian Thomas conceived the review, performed the literature search and analysis, drafted the manuscript and wrote and revised the manuscript.

## Funding

This work was supported by Deutsche Krebshilfe (grant #70117416).

## Ethics Statement

This review is based exclusively on previously published literature. It contains no new data from human participants, patient material, or animals, so ethical approval and informed consent were not required.

## Conflicts of Interest

The author declares no conflicts of interest.

## Data Availability

Data sharing not applicable to this article as no datasets were generated or analysed during the current study.
